# Comparative Transcriptome Profiling of the Early Infection of Wheat Roots by *Gaeumannomyces graminis* var. *tritici*

**DOI:** 10.1371/journal.pone.0120691

**Published:** 2015-04-14

**Authors:** Lirong Yang, Lihua Xie, Baoguo Xue, Paul H. Goodwin, Xin Quan, Chuanlin Zheng, Taiguo Liu, Zhensheng Lei, Xiaojie Yang, Yueen Chao, Chao Wu

**Affiliations:** 1 Institute of Plant Protection Research, Henan Academy of Agricultural Sciences, Henan Key Laboratory for Control of Crop Diseases and Insect Pests, IPM Key Laboratory in Southern Part of North China for Ministry of Agriculture, Zhengzhou, P. R. China; 2 School of Environmental Sciences, University of Guelph, Guelph, Canada; 3 Institute of Plant Protection, Chinese Academy of Agricultural Sciences, Beijing, P. R. China; 4 Research Centre for Wheat, Henan Academy of Agricultural Science, Zhengzhou, P. R. China; 5 Economic Crop Research Institute, Henan Academy of Agricultural Science, Zhengzhou, P. R. China; Beijing Institute of Microbiology and Epidemiology, CHINA

## Abstract

Take-all, which is caused by the fungal pathogen, *Gaeumannomyces graminis* var. *tritici (Ggt)*, is an important soil-borne root rot disease of wheat occurring worldwide. However, the genetic basis of *Ggt* pathogenicity remains unclear. In this study, transcriptome sequencing for *Ggt* in axenic culture and *Ggt*-infected wheat roots was performed using Illumina paired-end sequencing. Approximately 2.62 and 7.76 Gb of clean reads were obtained, and 87% and 63% of the total reads were mapped to the *Ggt* genome for RNA extracted from *Ggt* in culture and infected roots, respectively. A total of 3,258 differentially expressed genes (DEGs) were identified with 2,107 (65%) being 2-fold up-regulated and 1,151 (35%) being 2-fold down-regulated between *Ggt* in culture and *Ggt* in infected wheat roots. Annotation of these DEGs revealed that many were associated with possible *Ggt* pathogenicity factors, such as genes for guanine nucleotide-binding protein alpha-2 subunit, cellulase, pectinase, xylanase, glucosidase, aspartic protease and gentisate 1, 2-dioxygenase. Twelve DEGs were analyzed for expression by qRT-PCR, and could be generally divided into those with high expression only early in infection, only late in infection and those that gradually increasing expression over time as root rot developed. This indicates that these possible pathogenicity factors may play roles during different stages of the interaction, such as signaling, plant cell wall degradation and responses to plant defense compounds. This is the first study to compare the transcriptomes of *Ggt* growing saprophytically in axenic cultures to it growing parasitically in infected wheat roots. As a result, new candidate pathogenicity factors have been identified, which can be further examined by gene knock-outs and other methods to assess their true role in the ability of *Ggt* to infect roots.

## Introduction

Global wheat production is severely affected by take-all, a major fungal disease that is caused by *Gaeumannomyces graminis* var. *tritici* (*Ggt*), which can lead to yield losses of up to 40%-60% [1]. *Ggt* is a necrotrophic pathogen infecting wheat roots via hyphae that can survive in the soil in root debris of wheat plants. *Ggt* hyphae penetrate root cortical cells causing a root rot and then progress into the base of the stem, disrupting water flow. The result is stunting and premature death of the plant with symptoms of white heads, empty spikes, reduced grain panicles and reduced grain weight. *Ggt* is highly invasive on wheat roots, but has a wide host range, including wheat, triticale, barley and rye [2, 3]. Hence, considerable effort has been exerted to understand the mechanisms underlying *Ggt* pathogenicity to help reduce *Ggt*-caused wheat losses [4–8].

The ability of a soil-borne fungus, like *Ggt*, to cause disease on roots is affected by many factors, such as how well it is able to colonize the root surface and infect roots with the use of extracellular enzymes, toxins, and effectors. In *Ggt*, some genes coding for extracellular enzymes have been linked to pathogenicity, such as laccases [7, 9], endo-β-1,4-xylanase [4], β-1,3-glucanase [5], β-1,3-exoglucanase [10] and gentisate 1,2-dioxygenase-like enzyme [10]. For another soil-borne fungus, *Fusarium*, some genes encoding for proteins of the signaling pathway, such as mitogen-activated protein kinase [11–12], vacuolar Ca^2+^ exchanger protein [13] and plasma membrane calcium ATPase, have also been implicated in pathogenesis through recognition and signal transduction of extracellular signals [14]

The mechanisms of pathogenicity of *Ggt* to wheat roots are still not well understood as most research on wheat–*Ggt* interactions has focused on biological characteristics of the disease [15], pathogen distribution [16], pathogen genetic diversity [17], and the use of antagonists [18]. One approach to elucidating the mechanisms of fungal plant pathogenicity is to conduct large scale transcipt sequencing (RNA-seq analysis) of infected plant tissues. An advantage of RNA-seq is that the level of detection of transcript abundance enables broad measurements of expression levels of transcripts without prior sequence knowledge. Recent RNA-seq studies on plant-pathogen interactions include *Magnaporthe oryzae* infection of rice [19] and *Fusarium graminearum* infection of barley [20]. However, there have not yet been any RNA-seq studies of *Ggt*-plant interactions. The present study is an RNA-seq examination of the transcriptomes of *Ggt* in axenic culture and *Ggt* in infected wheat roots using the Illumina GA IIx sequencing platform. This data was then used for de novo assembly of the reads into contigs, and then mapping the reads against the contigs to identify differentially expressed genes (DEGs) between the two conditions.

## Materials and Methods

### Plant and fungal material and infection of roots

An isolate of *Ggt* (GGT-007, Henan Academy of Agricultural Sciences, Zhengzhou, China) was isolated from wheat root samples, This strain was identified by morphological characteristics, pathogenicity and molecular identification according to the methods of Quan.et al. [62] Liquid cultures were grown in potato dextrose broth at 25°C with shaking at 180 rpm for 5 d to prepare inoculum for wheat root infection and *Ggt* RNA extraction.

Seeds of winter wheat (*Aestivum triticum* cv 'Zhengmai 366') were placed in 75% ethanol for 5 s, rinsed with sterile water three times, surface sterilized with 1% AgNO_3_ for 9 min, and then rinsed again with sterile water five times. The seeds were germinated in sterile Petri dishes (12 cm diameter) with sterile filter paper and 5 mL of sterile water. After one day, the germinated wheat seedlings were transferred to another sterile Petri dish with sterile filter paper (50 seedlings and 10 mL sterile water added per dish). After two days, the root lengths were 2 cm to 3 cm, and the seedlings were transferred to tissue culture vessels containing *Ggt* inoculum (5 mg mycelium in 5 mL potato dextrose broth). All plants were grown in 16 h day/8 h night at 22°C.

### Sampling and experimental design

Root samples of the control group from two biological replicates were collected at 1, 2, 3, 4, 5, 7, and 9 d after inoculation. The samples were immediately frozen and then stored in liquid nitrogen until analysis. Total RNA was extracted from these materials using RNAiso Plus (Total RNA extraction reagent) (TaKaRa, Otsu, Japan). RNA purity was verified using a NanoPhotometer spectrophotometer (Implen, Westlake Village, CA, USA). RNA concentration was measured using a Qubit RNA Assay Kit in a Qubit 2.0 Fluorometer (Life Technologies, Grand Island, NY, USA). RNA integrity was assessed using the RNA Nano 6000 Assay Kit of the Bioanalyzer 2100 system (Agilent Technologies, Wilmington, DE, USA).

### Library construction and sequencing for RNA-seq

Sequencing libraries were generated using 3 μg of RNA per sample with a NEBNext Ultra RNA Library Prep Kit for Illumina (NEB, Ipswich, MA, USA) following the manufacturer’s recommendations, and tags were added to each sample for identification. Briefly, mRNA was purified from total RNA using poly-T oligo-attached magnetic beads. Fragmentation was performed using divalent cations under elevated temperature in NEBNext First Strand Synthesis Reaction Buffer (5×). First-strand cDNA was synthesized using random hexamer primer and M-MuLV Reverse Transcriptase (RNase H-). Second-strand cDNA was synthesized using DNA polymerase I and RNase H. Remaining overhangs were converted into blunt ends via exonuclease/polymerase activities. After the adenylation of 3′ ends of DNA fragments, NEBNext Adaptor with hairpin loop structure was ligated to prepare for hybridization. To select cDNA fragments of preferentially 150 bp to 200 bp in length, the library fragments were purified with the AMPure XP system (Beckman Coulter, Beverly, MA, USA). Then, 3 μL of USER Enzyme (NEB, Ipswich, MA, USA) was used with size-selected, adaptor-ligated cDNA at 37°C for 15 min and then at 95°C for 5 min before PCR. PCR was performed with Phusion High-Fidelity DNA polymerase, Universal PCR primers, and Index (X) Primer. The PCR products were purified (AMPure XP System, Brea, CA, USA), and library quality was assessed on the Agilent Bioanalyzer 2100 system.

### Clustering and sequencing and quality control

The tag-coded samples were clustered on a cBot Cluster Generation System using the TruSeq PE Cluster Kit v3-cBot-HS (Illumia, San Diego, CA, USA) according to manufacturer’s instructions. After cluster generation, the library preparations were sequenced on an Illumina Hiseq 2000 platform to generate 100 bp paired-end reads were generated. Raw data (raw reads) of fastq format were initially processed through in-house PERL scripts provided by Novogene (China). In this step, the clean reads were obtained by removing adapter sequences, reads with more than 10% N, and low-quality sequences (more than 50% of the reads having aphred base sQ ≤ 5. The Q20, Q30, and GC contents of the clean data were calculated. All downstream analyses were based on the quality-trimmed clean data.

### Read mapping to the reference genome

*Ggt* genome and gene model annotation files were directly downloaded from the genome website (ftp://ftp.ensemblgenomes.org/pub/release-20/fungi/fasta/gaeumannomyces_graminis/dna/). Index of the reference genome was constructed using Bowtie v2.0.6 [63], and paired-end clean reads were aligned to the reference genome using TopHat v2.0.9 (http://tophat.cbcb.umd.edu/) [64] with all parameters set to their default values.

### Differential expression analysis

HTSeq v0.5.4p3 [65] was used to count the read numbers that were mapped to each gene. The Reads Per Kilobase of exon model per Million mapped reads (RPKM) of each gene was calculated based on the length of the gene and read counts that were mapped to this gene. RPKM simultaneously considers the effect of sequencing depth and gene length for the read counts, and it is currently the most commonly used method for estimating gene expression levels [22].

Differential expression analysis between *Ggt* in culture and *Ggt* in infected wheat roots was performed using the DESeq R package (1.10.1) using a model based on the negative binomial distribution [66]. The resulting P-values were adjusted using the Benjamini and Hochberg’s approach for controlling the false discovery rate. Genes with an adjusted P-value <0.05 found by DESeq were assigned as differentially expressed.

### Gene Ontology (GO) and Kyoto Encyclopedia of Genes and Genomes (KEGG) enrichment analysis, novel transcript prediction, and alternative splicing analysis

GO enrichment analysis of DEGs was implemented by the GO seq R package [67], in which gene length bias was corrected. GO terms with corrected P value less than 0.05 were considered significantly enriched by DEGs. KEGG is a database resource for understanding high-level functions and utilities of the biological system [68], such as the cell, the organism, and the ecosystem, from molecular-level information, particularly large-scale molecular datasets generated by genome sequencing and other high-throughput experimental technologies (http://www.genome.jp/kegg/). KOBAS software was used to test the statistical enrichment of DEGs in KEGG pathways. The Cufflinks v2.1.1 Reference Annotation Based Transcript assembly method was used to construct and identify both known and novel transcripts from TopHat alignment results. Alternative splicing events were classified into 12 basic types by Asprofile v1.0. The number of AS events in each sample was estimated.

### Quantitative real-time PCR (qRT-PCR) validation

The expression levels of 12 DEGs were determined by qRT-PCR to confirm the results of mRNA-Seq analysis. Total RNA (1 μg) from *Ggt* culture (5 d after inoculation) and *Ggt*-infected wheat roots (1, 2, 3, 4, 5, 7, and 9 d after inoculation) was reverse transcribed using a PrimeScript RT reagent Kit with gDNA Eraser (Perfect Real Time) (Takara, Otsu, Japan) for RT-PCR according to the manufacturer’s protocols. qRT-PCR was performed on the Step One Plus Real-Time PCR System (Applied Biosystems, Foster City, CA, USA) with SYBR Premix Ex Taq (Tli RNaseH Plus, Takara, Otsu, Japan). The qRT-PCR conditions were as follows: 95°C for 30 s and 40 cycles of 95°C for 5 s and 60°C for 5 s. Then, melting curves were generated. The primers used in qRT-PCR for DEG validation are shown in S1 Table. Each plate was repeated thrice in independent runs for all reference and selected genes. Gene expression was evaluated by the 2^-ΔΔ Ct^ method [69].

## Results and Discussion

### mRNA-Seq general data analyses

RNA-seq expression profiling of *Ggt* was performed under two conditions: *Ggt* cultured in potato dextrose broth (PDB) and symptomatic *Ggt*-infected wheat roots. For pure cultures of *Ggt* in PDB, the fungus was grown for 5 days as that is the time period used to prepare inoculum. Growing *Ggt* for longer periods in PDB does not increase the level of disease when hyphae is used to inoculated plants. The samples for *Ggt*-infected wheat roots were chosen at 7 days post-inoculation when symptoms of root rot were prevalent but tissue damage was not so severe and so extraction of high quality RNA was still possible. One biological replicate with paired-end was sequenced for each condition. A total of 30,305,754 and 83,222,108 raw reads resulting in 26,172,744 and 77,645,702 clean reads were generated from the RNA of *Ggt* in culture and *Ggt*-infected wheat roots, respectively (Table 1). The quality of each library was similar with 95.86% and 94.88% of the raw reads from the *Ggt* culture and *Ggt*-infected wheat roots having quality values of Q≥20 and error probabilities of 0.05 and 0.06, respectively. The GC contents were almost identical for the *Ggt* and *Ggt*-infected wheat roots (59.71% and 58.30%, respectively).

**Table 1:** Transcriptome statistics of cDNA libraries from 5 day old culture of *Ggt* and 7 day old *Ggt*-infected wheat roots.

Sample name	Raw reads^1^	Clean reads^2^	Cleanbases^3^	Error rate^4^(%)	Q20^5^ (%)	Q30^6^ (%)	GC content^7^ (%)
*Ggt* culture	30,305,754	26,172,744	2.62G	0.05	96.62	87.83	59.71
*Ggt*-infected wheat roots	83,222,108	77,645,702	7.76G	0.05	95.59	87	58.39

^1^ The numbers of original data sequence

^2^ The filtered data sequence

^3^ *Qphred* = - 10*log*_10_(*e*)

^4^ the sequence length multiplied by the number of sequencing

^5^ The percentage of bases with a Phred value>20

^6^ The percentage of bases with a Phred value>30

^7^ The percentage of bases number of G and C

The sequenced reads were mapped to the *Ggt* genome. Among the transcripts from the *Ggt* culture and *Ggt*-infected wheat roots, up to 87% and 63%, respectively, of the total reads (52,345,488 and 155,291,404) were uniquely mapped to the *Ggt* genome, whereas only small proportions (0.19% and 0.13%) were mapped to multiple locations in the *Ggt* genome (Table 2). Among the uniquely mapped reads, 94.5% were mapped to the genome over one exon, 5.1% over an intergenic region, and 0.4% over an intron. Reads mapped to intergenic regions occurred because some gene annotations were inadequate. All uniquely mapped reads were used to calculate reads per kilobase of exon model per million mapped reads (RPKM) values, which were used to normalize expression levels. Alternative splicing (AS) events had occurred for 24,498 uniquely mapped reads from the *Ggt* culture and 23,410 uniquely mapped reads from the *Ggt*-infected wheat, and these were classified into 12 types using Asprofile v1.0 (S2 Table). The largest AS event groups under both conditions were TSS (alternative 5’ first exon) and TTS (alternative 3’ last exon) events.

**Table 2:** Summary of mapping the sequenced reads to the *Ggt* genome from the 5 day old culture of *Ggt* and 7 day old *Ggt*-infected wheat roots.

Event of mapping	Sample	
	*Ggt* culture	*Ggt*-infected wheat roots
Total reads	52,345,488	155,291,404
Total mapped^1^	45,808,804 (87.51%)	98,785,653 (63.61%)
Multiple mapped^2^	96,928 (0.19%)	205,702 (0.13%)
Uniquely mapped^3^	45,711,876 (87.33%)	98,579,951 (63.48%)
Read-1^4^	22,935,163 (43.81%)	49,503,896 (31.88%)
Read-2^5^	22,776,713 (43.51%)	49,076,055 (31.6%)
Reads map to '+'^6^	22,844,490 (43.64%)	49,230,953 (31.7%)
Reads map to '-'^7^	22,867,386 (43.69%)	49,348,998 (31.78%)
Non-splice reads	39,686,714 (75.82%)	86,211,659 (55.52%)
Splice reads	6,025,162 (11.51%)	12,368,292 (7.96%)

^1^Total number of reads mapped on the *Ggt* genome

^2^Total number of reads mapped to multiple locations in *Ggt* genome

^3^Total number of reads mapped to uniquely locations in the *Ggt* genome

^4^ The two directions of the paired-end sequencing

^5^ The two directions of the paired-end sequencing

^6^Total number of reads mapped to positive strand of *Ggt* genome

^7^ Total number of reads mapped to negative strand of *Ggt* genome

### Identification of differentially expressed genes (DEGs) between *Ggt* growing in culture and wheat roots

**Fig 1:**
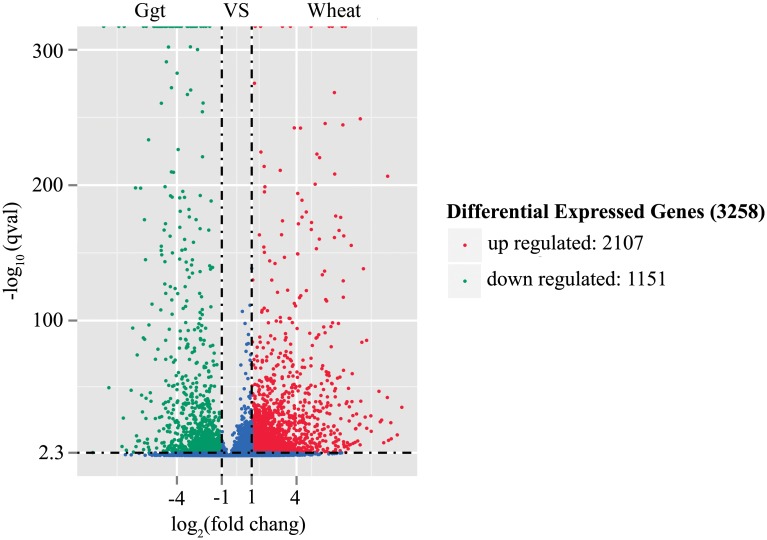
Volcano of DEGs between *Ggt* culture and *Ggt*-infected wheat roots. Ggt: *Ggt*-culture, wheat: *Ggt*-infected wheat roots; the y-axis corresponds to the mean expression value of log10 (p-value), and the x-axis displays the log2 fold change value. The red dots represent up-regulated DEGs, the blue dots represent down-regulated DEGs.

Log 2-fold DEGs between *Ggt* culture and *Ggt*-infected wheat roots were identified using DEG-Seq, and P-values were corrected by the Hochberg and Benjamini method [21]. Corrected P-value of 0.005 and log2 (fold change) ±1 were set as thresholds for significant differential expression. A total of 3,258 DEGs were detected between the *Ggt* culture and infected wheat root libraries, with 2,107 up-regulated genes and 1,151 down-regulated genes in the *Ggt*-infected roots compared to the *Ggt* culture (Fig 1).

The DEGs were divided into three groups according the RPKM values as per Mortazavi et al. [22]. Genes with RPKM values between 0 to 3 were considered to be expressed at a low level, 3 to 15 were at a medium level and above 15 were at a high level (Table 3). The percentage of highly expressed genes was smaller in the control than in the *Ggt* samples, whereas the percentage of low-level expressed genes was larger in the *Ggt* culture than in the *Ggt*-infected root samples. The Pearson correlation coefficient for the replicates calculated by log^10^ RPKM was 1 and between the *Ggt* culture and infected samples was 0.71, indicating reliable sequencing data (S1 Fig). A comparison of the RPKM distribution of the DEGs showed that the box plots of the log10 (RPKM+1) values and distribution of the density of the log10 (RPKM+1) values overlapped between the samples from the *Ggt* culture and *Ggt*-infected roots indicating that the range of of the expression values in the two samples were generally similar, although there was a considerable spread in the expression levels of the DEGs (S2 Fig).

**Table 3:** Number of transcripts of *Ggt* culture and *Ggt*-infected wheat roots at different expression level intervals.

Sample	RPKM level				
	0~1	1~3	3~15	15~60	>60
*Ggt* culture	4068(26.90%)	2046(13.53%)	4337(28.67%)	2944(19.46%)	1730(11.44%)
*Ggt*-infected wheat roots	3462(22.89%)	1448(9.57%)	3755(24.83%)	4402(29.10%)	2058(13.61%)

RPKM levels were chosen based on divisions described in [22]

**Fig 2:**
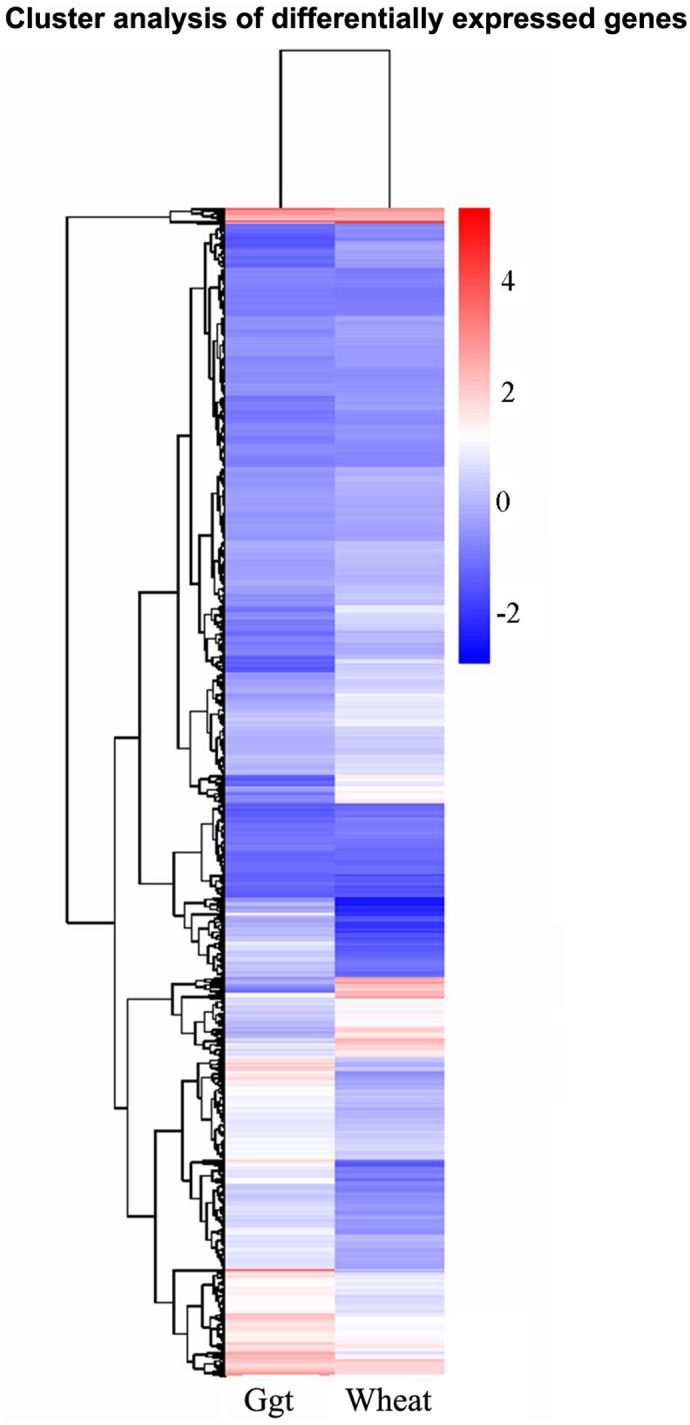
Hierarchical clustering analyses of DEGs. According to the two sample’s log10 (RPKM+1), red indicates high expression of the gene, blue indicates low expression of genes. Ggt: *Ggt*-cultures, Wheat: *Ggt*-infected wheat roots.

Hierarchical clustering of the DEGs according to the log10 (RPKM+1) values showed the overall gene expression pattern to be divided into several clusters based on the expression levels of the DEGs in *Ggt* culture versus *Ggt*-infected roots conditions (Fig 2). Only one relatively small cluster contained DEGs with very high expression levels in both *Ggt* culture and *Ggt*-infected roots, whereas all the other DEGs formed a second cluster with multiple subclusters mostly showing medium to low expression levels under both conditions or slightly higher expression under either the *Ggt* culture or *Ggt*-infected root conditions.

### Functional annotation and classification of DEGs

**Fig 3:**
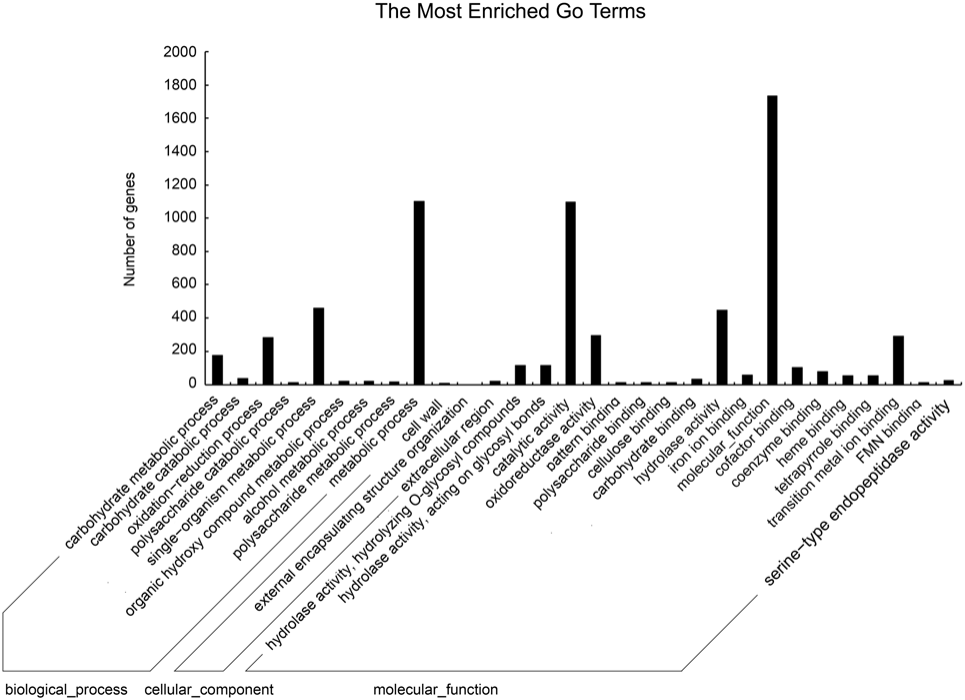
Functional annotation of DEGs based on gene ontology (GO) categorization. Each annotated sequence was assigned at least one GO term.

The GOseq R package was used to annotate and assign different functional GO categories to the DEGs of *Ggt* in infected wheat roots. The 3,258 significant DEGs between the *Ggt* culture and *Ggt*-infected wheat roots (q-value < 0.05) belonged to 9 GO groups based on biological process, 3 groups based on cellular component and 18 groups based on molecular function (Fig 3). For biological process, the dominant categories were metabolism process (GO: 0008152) with 1,101 DEGs, single-organism metabolism (GO: 0044710) with 461 DEGs, oxidation-reduction processes (GO: 0055114) with 285 DEGs and carbohydrate metabolic processes (GO: 0005975) with 176 DEGs. For cellular component, the three categories were extracellular region (GO: 0005576) with 25 DEGs, external encapsulating structure (GO: 0030312) with 14 DEGs and cell wall (GO: 0005618) with 12 DEGs. For molecular function, the largest categories were general molecular function (GO: 0008152) with 1,733 DEGs, catalytic activity (GO: 0003824) with 1,099 DEGs, hydrolase activity (GO: 0016787) with.447 DEGs and transition metal ion binding (GO: 0046914) with 291 DEGs (Fig 3).

**Fig 4:**
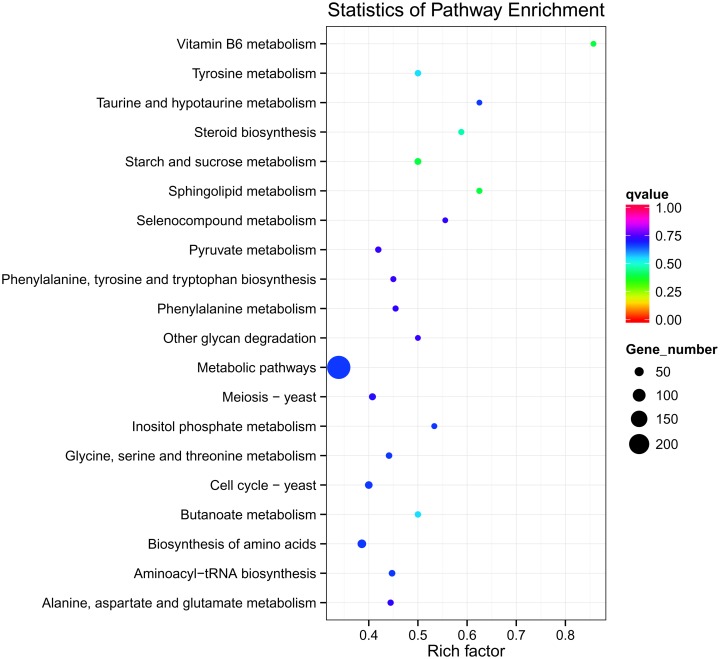
Statistics of KEGG pathway enrichment. The y-axis corresponds to KEGG Pathway, and the x-axis shows the enrichment factor. The color of the dot represent q value, and the size of the dot represents the number of DEGs mapped to the reference pathways.

The biological pathways of the DEGs of *Ggt* were mapped to the reference pathways in KEGG (http://www.genome.ad.jp/kegg/) [23]. The DEGs between the *Ggt* culture and *Ggt*-infected wheat roots were assigned to 100 KEGG pathways (Fig 4). The pathways with the most significant representation were general metabolic pathways (mgr01100) with 242 members and biosynthesis of secondary metabolites (mgr01110) with 100 members. These results indicate that fungal genes involved in the metabolism or biosynthesis of secondary metabolic pathways were being expressed more when the fungus was growing parasitically in wheat roots than growing saprophytically on culture medium, which may indicate an importance in the pathogenicity of *Ggt*.

### Expression of selected DEGs

**Fig 5:**
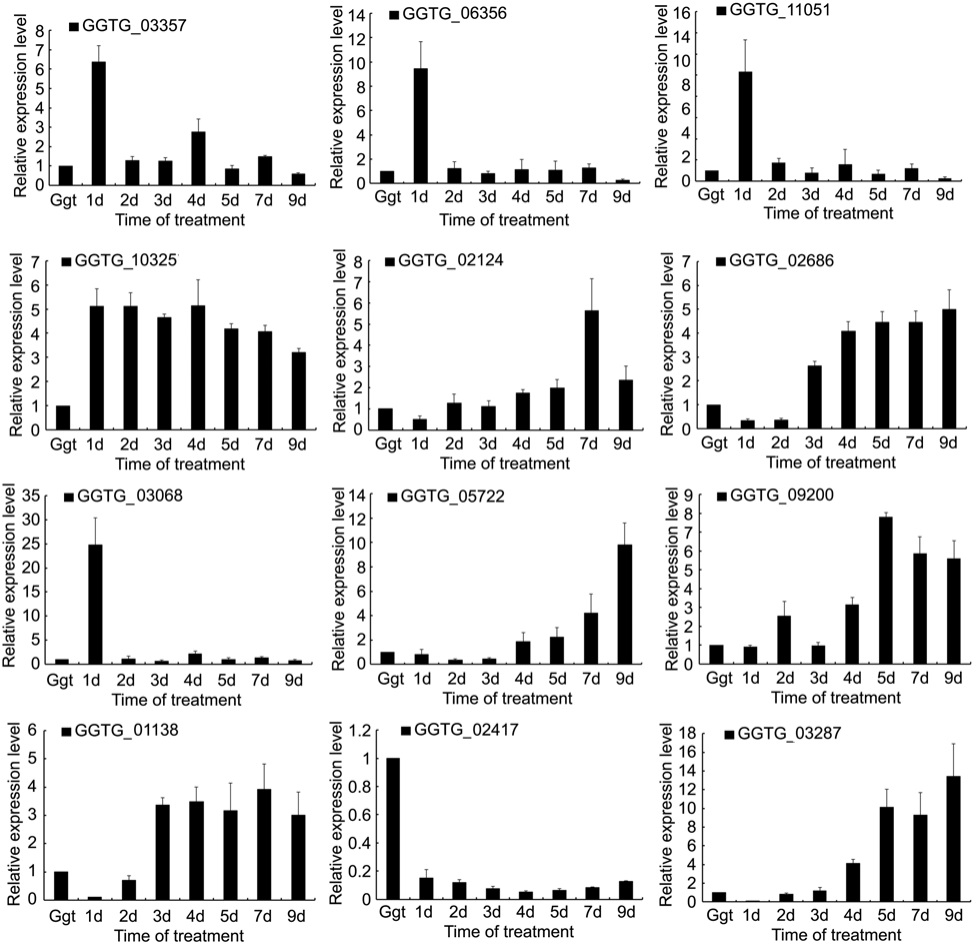
Relative expressions of selected DEGs in *Ggt* culture and *Ggt*-infected wheat roots. The x-axis shows the sample: *Ggt* culture, and infected wheat roots at 1 day, 2 days, 3 days, 4 days, 5 days, 7 days and 9 days post infection.

qRT-PCR of 12 selected DEGs was performed to validate the RNA-seq data. Eleven were up-regulated DEGs and one was a down-regulated DEG between *Ggt* culture and *Ggt*-infected wheat roots (Fig 5). A comparison of the ratio of the qRT-PCR expression and RPKM values for the *Ggt* culture to the 7 day *Ggt*-infected wheat roots revealed that these were basically consistent indicating that the RNA-seq data were credible (Table 4).

**Table 4:** The ratio of the expression levels of 12 DGEs of 7 day old *Ggt*-infected wheat roots to *Ggt* 5 days in culture as determined by qRT-PCR versus RPKM values from RNA-seq.

DEG number	qRT-PCR^1^	RPKM^2^
GGTG_06356	1.30	1.91
GGTG_10325	4.06	3.64
GGTG_03068	1.31	1.98
GGTG_03357	1.45	2.04
GGTG_11051	1.22	1.24
GGTG_01138	3.92	3.06
GGTG_03287	9.31	8.52
GGTG_02124	5.65	4.20
GGTG_02417	0.08	0.09
GGTG_05722	4.11	4.62
GGTG_02686	4.45	4.80
GGTG_09200	5.87	5.31

^1^ qRT-PCR ratio was determined from the log2 of the^− ΔΔ^ CT values from the *Ggt*-infected wheat root sample divided by that from the *Ggt* in culture

^2^ RPKM ratio determined by the log2 of the RPKM value from the *Ggt*-infected wheat roots divided by that of the *Ggt* culture sample.

qRT-PCR of the 11 selected up-regulated DEGs showed several patterns over the course of infection. Expression of GGTG_03357 (Adhesion and hyphal regulator 1), GGTG_06356 (Adenylate cyclase), GGTG_11051 (Calcium-binding protein) and GGTG_03068 (Protein scd2/ral3) were all relatively high at 1 day of the infection followed by much lower levels for the rest of the infection comparable to that of *Ggt* in culture. This indicates that these DEGs may be particularly important for penetration or establishing infection. Expression of GGTG_02124 (Glucan 1,3-beta-glucosidase), GGTG_02686 (Linoleate 9S-lipoxygenase), GGTG_05722 (Xyloglucan-specific endo-beta-1,4-glucanase), GGTG_09200 (Exopolygalacturonase B), GGTG_01138 (Scytalone dehydratase) and GGTG_03287 (Endo-1,4-beta-xylanase A) were more highly expressed late in the infection indicating roles in the later stages of root colonization. However, those DEGs did not all have the same pattern of change over time with some, like GGTG_02124 (Glucan 1,3-beta-glucosidase) and GGTG_01138 (Scytalone dehydratase), generally gradually increasing over time, while others, like GGTG_03287 (Endo-1,4-beta-xylanase A), being only be expressed for a limited time late in infection. Expression of GGTG_10325 (G2-specific protein kinase) was unique among the 11 selected up-regulated DEGs in that it remained relatively high throughout the infection compared to expression in culture, only slightly decreasing over time as the infection progressed. qRT-PCR of the one selected down-regulated DEG, GGTG_02417(Laccase-2), showed that its expression was relatively unchanged during infection with a slight decline in the mid-period of infection.

### DEGs for signal transduction pathways

The signal transduction pathway in plant pathogenic fungi is essential for surface recognition, adaptation to the host milieu, appresorium formation, infection establishment, and invasive growth [24–33]. Several DEGs were identified from the GO annotation or KEGG analyses related to Ca^2+^ signaling, cyclic AMP- protein kinase A (cAMP-PKA), and mitogen-activated protein kinase (MAPK) pathways.

Among the up-regulated DEGs related to the Ca^2+^ signaling pathway in *Ggt*- infected roots versus *Ggt* culture, there were two DEGs (GGTG_00202 and GGTG_02953) for vacuolar calcium ion transporter, three DEGs (GGTG_03594, GGTG_08053 and GGTG_08581) for calcium-transporting ATPase, two DEGs (GGTG_04060 and GGTG_08412) for calcium channel protein, and one DEG (GGTG_11051) for calcium-binding protein (S3 Table). In contrast, there was only one down-regulated DEG in the infected roots related to Ca^2+^ signaling (GGTG_02953) for vacuolar calcium ion transporter. Calcium ions are extremely important for signal transduction. Two important calcium mediators in eukaryotic cells are calmodulin and phosphatase, calcineurin. Calcineurin is required for fundamental biological events of pathogenic fungus, such as mating, morphogenesis and virulence [24–26]. Calcium transporters, such as vacuolar Ca^2+^ exchanger (*Vcx1*), calcium-channel protein *(Cch1*), and plasma membrane calcium ATPase (*Pmc1*), are required for fungal virulence, supporting a role for calcium-mediated signaling in fungal pathogenesis [27]. For example, a knockout of the *Ggt Vcx1* significantly decreased the pathogenicity of *Ggt* to wheat roots [6], a knockout of *PmcA* significantly reduced *Aspergillus fumigatus* virulence in invasive pulmonary aspergillosis of mice and a knockout of *PmcA* affected cation homeostasis and in cell wall integrity of *A*. *fumigatus* [14]. qRT-PCR of the DEG (GGTG_11051) for calcium-binding protein showed high expression early in infection (Fig 5). Ca^2+^ signaling may be more important for *Ggt* in infected roots than in *Ggt* culture because the fungus may need to identify and the change in the environment, particularly early in the infection when switching from saprophytic growth in the soil to parasitic growth inside wheat roots.

Among the up-regulated DEGs related to the cAMP-PKA pathway in *Ggt*-infected roots versus *Ggt* in culture, there was one DEG (GGTG_02473) for guanine nucleotide-binding protein alpha-2 subunit, one DEG (GGTG_06356) for adenylate cyclase, and one DEG (GGTG_05905) for cAMP-independent regulatory protein. qRT-PCR analysis showed that GGTG_06356 expression peaked at 1 d post-infection, and then decreased to the level observed in *Ggt* culture (Fig 5). The key components of the cAMP—PKA pathway include adenylate cyclase and regulatory and catalytic subunits of protein kinase A. Both small GTPase Ras and guanine nucleotide-binding protein alpha-2 subunit function upstream from the cAMP—PKA pathway. Adenylate cyclase is activated by Gα subunits in *Schizosaccharomyces pombe* and in the model filamentous fungus *Neurospora crassa* [28]. Once again, it appears that parasitic growth of *Ggt* may involve different signaling than saprophytic growth.

For the MAPK pathway, there were 8 DEGs up-regulated for eight key enzymes in the pathway (KEGG PATH: mgr04011, http://www.genome.jp/kegg/) in *Ggt*-infected roots versus *Ggt* culture. These were a DEG (GGTG_03068) for protein scd2/ral3, a DEG (GGTG_07051) for osmosensing histidine protein kinase, a DEG (GGTG_12416) for Rho guanine nucleotide exchange factor scd1, a DEG (GGTG_07905) for cytokinesis protein sepA, a DEG (GGTG_04689) for GTP-binding protein rho5, a DEG (GGTG_05786) for MAP kinase kinase kinase mkh1, a DEG (GGTG_10157) for MAP kinase kinase kinase wis4 and a DEG (GGTG_03934) for tyrosine-protein phosphatase pmp1. The MAPK pathway is highly conserved in eukaryotes from yeasts to humans [29, 30]. In this pathway, MAP kinase kinases kinases (MAPKKK) first activate MAP kinase kinases (MAPKK), which then activate MAP kinases (MAPK). The MAPK pathway in several fungal pathogens are well known for transducing various extracellular signals in regulating cell growth, differentiation, and condition, which are important for fungal pathogenesis [30–33]. qRT-PCR for GGTG_03068 showed that its expression was high at 1 d post-infection and then decreased to levels similar to that in *Ggt* cultures for the remainder of the infection (Fig 5). The scd2/ral3 protein has been shown to involved MAPK pathway in yeast during cell growth [29,31]. This indicates that the *Ggt* scd2/ral3-honolog may be needed for extracellular signal transduction in early root infection, such as during penetration. However, like the other DEGs in the signal transduction pathway of *Ggt*, parasitic growth in roots is a complex process that involves numerous factors, and RNA-seq only indicates its involvement. Additional studies on molecular and proteomic analysis are required to validate these predictions.

### DEGs for development

Several up-regulated DEGs were also found that have previously been related to development in fungal plant pathogens, some of which are directly linked to signaling pathways. There was one DEG for adhesion and hyphal regulator 1 (GGTG_03357), one DEG for scytalone dehydratase (GGTG_01138), one DEG for linoleate 9S-lipoxygenase (GGTG_02686), one DEG for DN24 (GGTG_03133), two DEGs for cyclophilin (GGTG_01246 and GGTG_06971), two DEGs for chitin synthase (GGTG 03012 and GGTG_14037), six DEGs for hydrophobin (GGTG_03085, GGTG_02383, GGTG_06272, GGTG_07637, GGTG_04864 and GGTG_08655), and six DEGs for phosphodiesterase (GGTG_01358, GGTG_01857, GGTG_03142, GGTG_06261, GGTG_10058 and GGTG_11065). Adhesion and hyphal regulator 1 is involved in cellular processes that are mediated through an iron-independent mechanism during development [34]. Scytalone dehydratase is an enzyme involved in the synthesis of dihydroxynapthalene-derived melanin, and it has been identified as a pathogenicity determinant of *M*. *grisea* [35]. Linoleate 9S- lipoxygenase of fungi may form specific oxylipins and participate in sporulation [36]. DN24, which is associated with nitrogen starvation, was expressed during infection of *Colletotrichum gloeosporioides* and was needed for normal hyphal development [37]. Cylcophilins are related to the signaling molecule, calcineurin, and a cyclophilin of *M*. *grisea* was linked to pathogenicity, appressorial development and hyphal growth [38]. Different chitin synthase genes of *M*. *oryzae* had different effects on virulence, appressorium penetration, hyphal growth and conidiation during infection [39]. A hydrophobin of *M*. *girsea* affected conidial production, conidial germination, appressorium formation and the ability to infect rice [40]. Phophodiesterases play roles in cAMP signaling affecting conidial morphology, cell wall integrity and pathogenicity of *M*. *oryzae* [41]. qRT-PCR was performed for three DEGs related to fungal development. The DEG for adhesion and hyphal regulator showed high expression early in the infection indicating a role in penetration and infection establishment. In contrast, qRT-PCR of a scytalone dehydratase DEG and a linoleate 9S- lipoxygenase DEG showed that both were highly expressed late in the infection indicating that melanin and oxylipin synthesis is needed later in the infection process.

### DEGs for plant cell wall degradation

A total of 62 DEGs were related to plant cell-wall-degrading-enzymes (CWDEs) in this study (S2 Table). There were 27 DEGs for cellulase, 12 for xylanase, 1 for xyloglucanase, 21 for glucosidase, 2 for pectinase and 1 for aspartic protease. CWDEs are needed for initial penetration, invasion within the host tissue and conversion of the host tissues into nutrients [42–44]. Plant cell walls are composed of pectin, cellulose, hemicelluloses and associated proteins [43]. Polygalacturonase can degrade pectin, which is the major sugar of the middle lamellae, resulting in rotting of the tissues, and an endopolygalacturonase was needed by *Botrytis cinerea* to grow in host tissue from the inoculation site [45]. Pectinesterase catalyzes the de-esterification of pectin into pectate and methanol and is also important in the virulence of *Botrytis cinerea* [44]. Cellulase hydrolyzes the β-1, 4 glycoside bonds in the cellulose polymer and have been implicated in the virulence of *B*. *cinerea* [46]. The hemicellulase, endo-β-1, 4-xylanase, hydrolyzes the β-1, 4-linked polysaccharide backbone of xylan, which forms the major component of hemicellulose, and among the three xylanases of *B*. *cinerea*, xyn11A is required for full virulence to tomato [47]. There are also a variety of proteins in plant cell walls with most having structural functions cross-linking in the cell wall, although some act in plant morphogenesis and development [48]. Aspartic proteases, possibly degrading some of structural proteins thus destabilizing cell wall integrity, have been implicated in the virulence of *B*. *cinerea*, although single and double gene knock-outs of five aspartic proteases did not affect pathogenicity [49]. qRT-PCR of two CWDEs, endo-1,4-beta-xylanase A (GGTG_03287) and exopolygalacturonase B (GGTG_09200), showed that both had was relatively low expression in culture and during infection, except at 7 d post-infection (Fig 5). This indicates that thes CWDEs may only be needed late in infection by *Ggt*, perhaps because of a need to degrade pectin and hemicellulose to obtain nutrients or to weaken the wall to allow hyphal growth in the root. If correct, then particular CWDEs may have highly specialized functions in the infection process of *Ggt*.

### DEGs for response to plant defense compounds

Roots have a variety of defense mechanisms against fungal pathogens, such as pathogenesis-related proteins and cell wall strengthening [50]. Callose is a cell wall material composed of β-1,3-glucan produced in response to wounding, infection by pathogens and abiotic stresses, and it provides penetration resistance against pathogens [51]. Callose degradation occurs by 1, 3-β-glucanase, which has been shown to be secreted by *Ggt* to break down post-infectionally [9]. Lignin is composed of hydroxycinnamyl alcohols (or monolignols), coniferyl alcohol and sinapyl alcohol and also helps to strengthen plant cell walls against microbial degradation [52]. Gentisate 1, 2-dioxygenase-like enzymes may be involved in lignin degradation by causing oxidative ring-opening of protocatechuate allowing lignin derived aromatic compounds to be degraded to phenols [53]. Laccases can also participate in lignin depolymerization by oxidizing phenolic and non-phenolic components of lignin [54]. There are three laccase genes (*Lac*1, *Lac*2, and *Lac*3) in the *Ggt* genome, and they play an important function in *Ggt* pathogenesis [9, 55]. In this study, two DEGs for laccase were up-regulated, while one was down-regulated. The DEGs associated with degradation of cell wall callose and lignin in this study imply a defense against plant defenses related to cell wall strengthening during *Ggt* growth inside wheat roots.

*Ggt* infection can induce the biosynthesis of phytoalexins in wheat [56]. The major phytoalexins in wheat are cyclic hydroxamic acids and 2.4-dihydroxy-7-methoxy-2H-1.4-benzoxazin-3(4H)-one [57, 58]. ATP-binding cassette (ABC) transporters are involved in limiting the sensitivity of the fungus to phytoalexins [59]. ABC transporter can act as efflux pumps, providing resistance to a variety of metabolic poisons, such as when a phytoalexin enters the hyphae [60]. There were 3 DEGs for ATP-binding cassette up-regulated in infected roots.

## Conclusions

This study is the first to analyze the transcriptome of *Ggt* and infected wheat root using Illumina platform. A comparison of *Ggt* in culture to that in roots showed that there were 3,258 2-fold DEGs, of which twice as many were up-regulated than down-regulated. Some of these DEGs have previously been shown to be closely related to *Ggt* pathogenicity, but many have not been previously associated with *Ggt* and are promising candidates for further investigation. To the best our knowledge, this study is the first to use Illumina deep sequencing technology to compare the entire transcriptome of *Ggt* growing saprophytically in culture to *Ggt* grwoing parasitically in wheat roots. qRT-PCR of a small number of the DEGs showed that expression can be very specific to certain times during the infection, and some DEGs may play specific roles either early or late in the disease.

The use of fungal genomics and transcriptomics has started to make major contributions to identifying genes required for pathogenesis, such as those related to signaling, penetration, fungal nutrition and host colonization [61]. The results of this study has contributed to advancing our knowledge of this pathosystem by revealing that *Ggt* preferentially expresses a considerable number of genes during parasitic growth in roots to signal changes in its environment as it colonizes the root, to combat host resistance mechanisms related to cell wall appositions and antimicrobial compounds, as well as to degrade the plant cell walls, possibly both for obtaining nutrients and allowing for growth of its hyphae through the root tissues. Ultimately, targeting these candidate virulence genes by further analysis, such as gene disruption, will result in a better understanding of these virulence mechanisms of *Ggt*, which may lead to improving its control resulting in higher yields of wheat and other crop hosts of *Ggt* worldwide

## Supporting Information

S1 Fig The Pearson correlation coefficient between *Ggt* culture and *Ggt*-infected wheat roots calculated using log10-based RPKM. Ggt: *Ggt* culture, Wheat: *Ggt*-infected wheat roots.
(TIF)

S2 Fig Comparison of the RPKM distribution for DEGs between *Ggt* culture (*Ggt*) and *Ggt*-infected wheat roots (wheat). Fig.a: RPKM distribution with the y-axis displaying log10 (RPKM+1). Fig.b: RPKM density distribution with the x-axis displaying log10 (RPKM+1).
(TIF)

S1 Table The primers used in this study. (PDF)

S2 Table Alternative splicing (AS) events of *Ggt* culture and *Ggt*-infected wheat roots. (PDF)

S3 Table DEGs for signal transduction pathways, plant cell wall degradation and response to plant defense compounds. (PDF)
