# Supplementary figures and images for: Comparative Transcriptome Profiling of the Early Infection of Wheat Roots by Gaeumannomyces graminis var. tritici
Source: PLoS One. 2015 Apr 14;10(4):e0120691. doi: 10.1371/journal.pone.0120691 (PMC4397062; doi:10.1371/journal.pone.0120691)

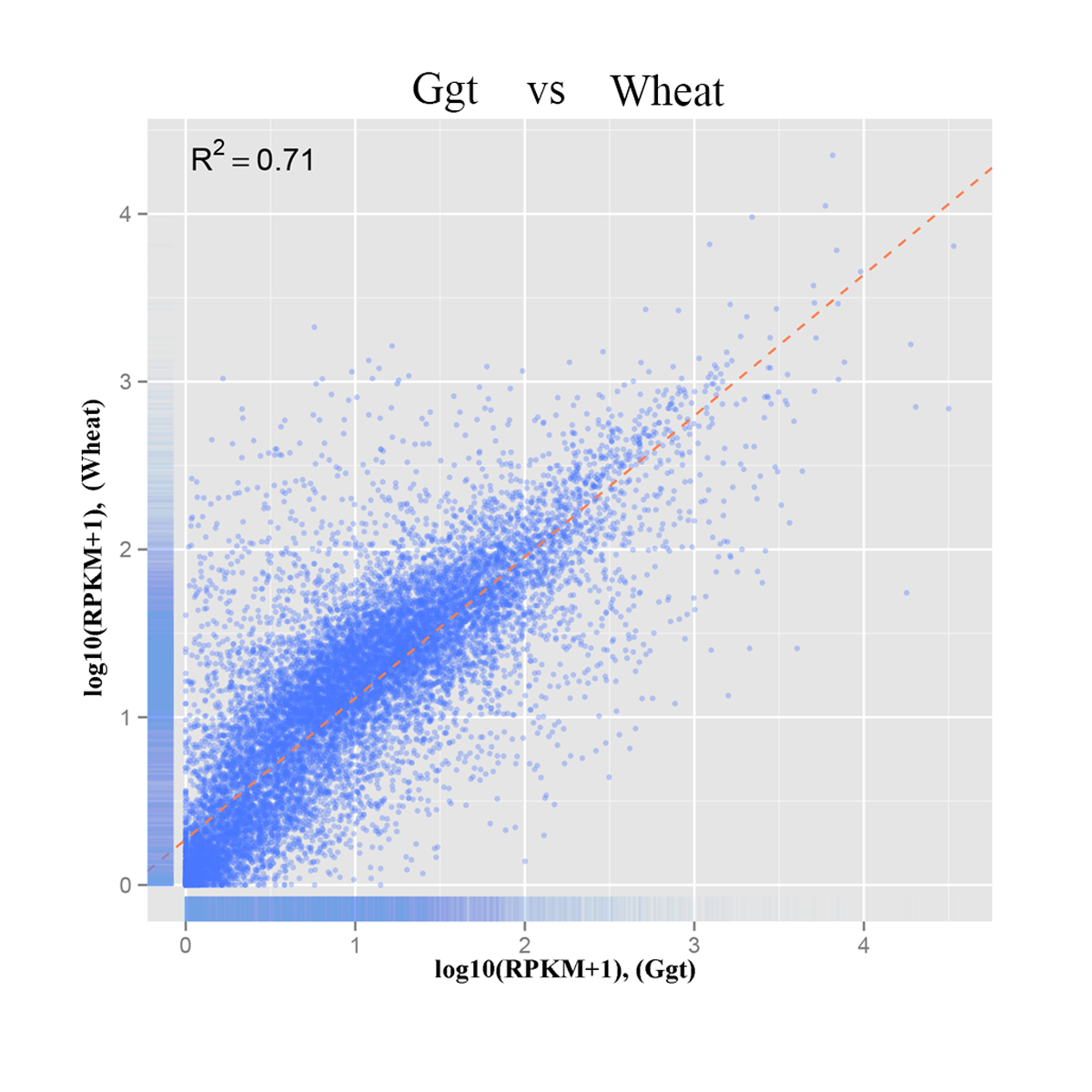

Supplement: S1 Fig — Ggt: Ggt culture, Wheat: Ggt-infected wheat roots. (TIF) [file pone.0120691.s001.tif]

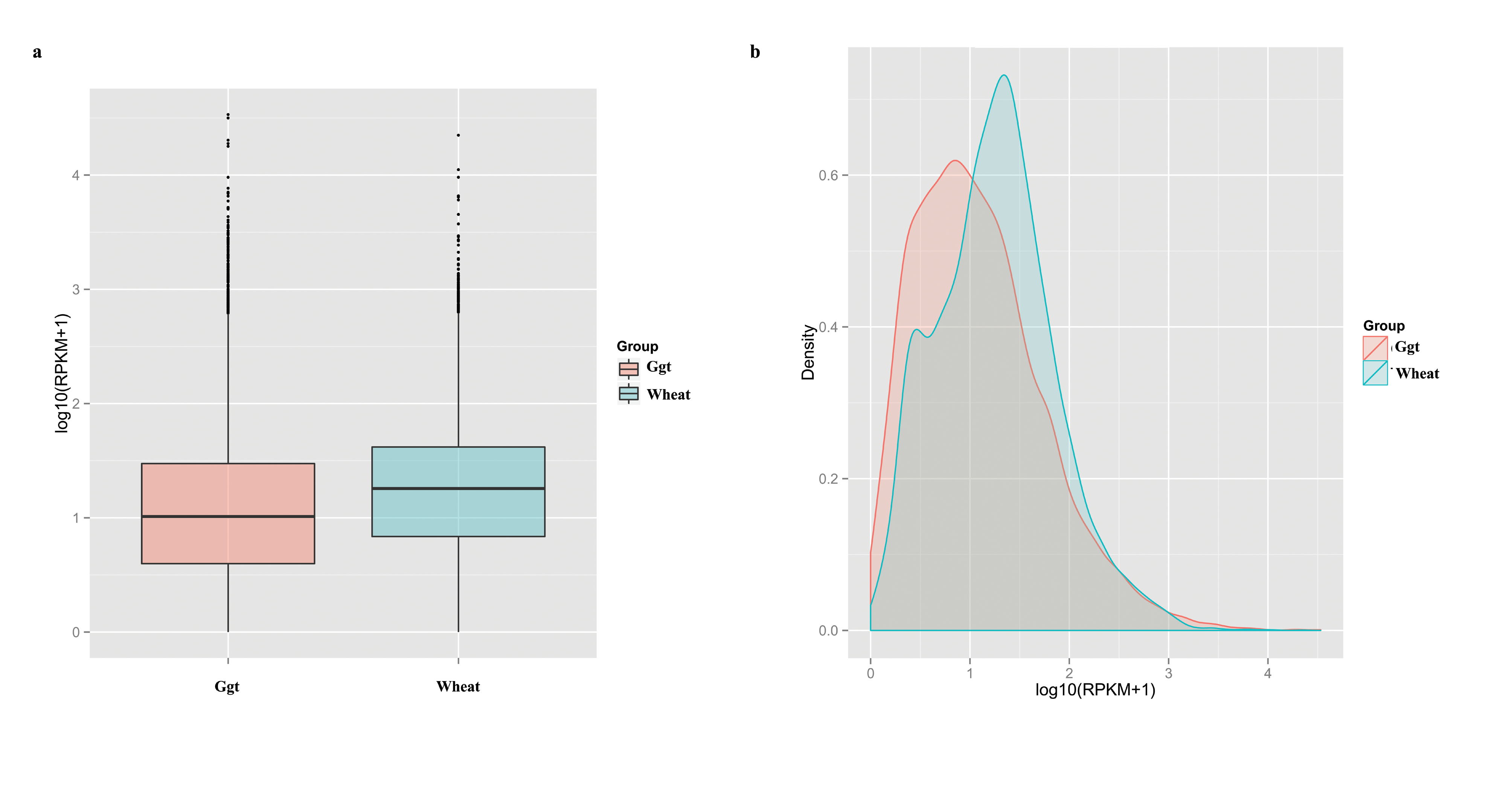

Supplement: S2 Fig — Fig.a: RPKM distribution with the y-axis displaying log10 (RPKM+1). Fig.b: RPKM density distribution with the x-axis displaying log10 (RPKM+1). (TIF) [file pone.0120691.s002.tif]
