# Supplementary material for: Comparative Transcriptome Profiling of the Early Infection of Wheat Roots by Gaeumannomyces graminis var. tritici
Source: PLoS One. 2015 Apr 14;10(4):e0120691. doi: 10.1371/journal.pone.0120691 (PMC4397062; doi:10.1371/journal.pone.0120691)
Supplement: S1 Table — (PDF) [file pone.0120691.s003.pdf]

S1 Table The primers used in this study.

Gene_id	Annotation	Forward primer	Reverse primer
GGTG_06356	Adenylate cyclase	TTAGGTTGTTGCCACGGACG	CAGTGTCGCCGATTCGTTCA
GGTG_10325	G2-specific protein kinase	GGAACCATGACCATCCTGCA	TACTTCTCCGCCGCACAAAT
GGTG_03068	Protein scd2/ral3	TTAGGTTGTTGCCACGGACG	CAGTGTCGCCGATTCGTTCA
GGTG_03357	Adhesion and hyphal regulator 1	TGTCCATCCACACACCCACT	CAATACAAGGCCAGCGACG
GGTG_11051	Calcium-binding protein	TGCCGGATCAGATACATGGC	GTTCGACCAGGAGTACGCAA
GGTG_01138	Scytalone dehydratase	TACCGCTCGTTCCTGGACAA	GGCGTCGGTGTACTTTTGGT
GGTG_03287	Endo-1,4-beta-xylanase A	AGGGCAAGTGTTACGCATGG	TTCGTCTTTGCGCCGTTGTA
GGTG_02124	1,3-beta-glucosidase	CTGCCTGCGACCCAAATCAT	GTCGTGCGATGCTTCCGTTA
GGTG_02417	Laccase-2	ACTTCTCGCTGCAGTACACC	TTGGTGTCGGTGCTGTTCTT
GGTG_05722	Xyloglucan-beta-1,4-glucanase	CAGCGTCAAGTCGTACTCCA	CAGCCACACCATGATCTCGT
GGTG_02686	Linoleate 9S-lipoxygenase	TGAGCCTCACCAAGCAGAAG	GACAGCGTCTTTTGCCAGTG
GGTG_09200	Exopolygalacturonase B	TCGACGACTTTCAGCTGCAC	CCGGCAAACTCATTCCACCA
