# Supplementary material for: Comparative Transcriptome Profiling of the Early Infection of Wheat Roots by Gaeumannomyces graminis var. tritici
Source: PLoS One. 2015 Apr 14;10(4):e0120691. doi: 10.1371/journal.pone.0120691 (PMC4397062; doi:10.1371/journal.pone.0120691)
Supplement: S2 Table — (PDF) [file pone.0120691.s004.pdf]

S2 Table Alternative splicing (AS) events of *Ggt* culture and *Ggt*-infected wheat roots.

AS event	Number of mapped reads	
	Ggt-infected wheat roots	*Ggt*-cultures
TSS	10,393	10,831
TTS	10,329	10,745
SKIP	62	76
XSKIP	66	78
MSKIP	6	8
XMSKIP	4	10
IR	678	782
XIR	1026	1076
MIR	108	108
XMIR	226	240
AE	357	367
XAE	155	177

The types of AS events are: TSS: alternative splicing of the first exon at transcription start site ; TTS: alternative splicing of last exon at transcription terminal site, SKIP: exon skipping (in this case, an exon may be spliced out of the primary transcript or retained); XSKIP: approximate exon skipping (XSKIP_ON,XSKIP_OFF pair); MSKIP: multiple exon skipping (MSKIP_ON, MSKIP_OFF pair); XMSKIP: Approximate Multiple exon skipping (XMSKIP_ON,XMSKIP_OFF pair); IR: Intron retention (A sequence may be spliced out as an intron or simply retained); XIR: Approximate Intron retention (XIR_ON, XIR_OFF pair); MIR: Multiple Intron retention (MIR_ON, MIR_OFF pair); XMIR: Approximate Multiple Intron retention (XMIR_ON, XMIR_OFF pair); AE: Alternative exon ends (5’, 3’, or both); XAE: Approximate Alternative exon ends.
