# Supplementary material for: Comparative Transcriptome Profiling of the Early Infection of Wheat Roots by Gaeumannomyces graminis var. tritici
Source: PLoS One. 2015 Apr 14;10(4):e0120691. doi: 10.1371/journal.pone.0120691 (PMC4397062; doi:10.1371/journal.pone.0120691)
Supplement: S3 Table — (PDF) [file pone.0120691.s005.pdf]

S3 Table DEGs for signal transduction pathways, plant cell wall degradation and response to plant defense compounds.

Category	Annotation	DGE
Ca^{2+} signaling pathways	Vacuolar calcium ion transporter	GGTG_00202, GGTG_02953
	Calcium-transporting ATPase	GGTG_03594, GGTG_08053, GGTG_08581
	Calcium channel	GGTG_04060,GGTG_08412
	Calcium-binding protein	GGTG_11051
cAMP–PKA pathway	Guanine nucleotide-binding protein	GGTG_02473
	Adenylate cyclase	GGTG_06356
	cAMP-independent regulatory protein	GGTG_05905
MAPK pathway	Protein scd2/ral3	GGTG_03068
	Osmosensing histidine protein kinase	GGTG_07051
	G2-specific protein kinase	GGTG_10325
	Rho guanine nucleotide exchange factor scd1	GGTG_12416
	Cytokinesis protein sepA	GGTG_07905
	GTP-binding protein rho5	GGTG_04689
	MAP kinase kinase kinase mkh1	GGTG_05786
	MAP kinase kinase kinase wis4	GGTG_10157
	Tyrosine-protein phosphatase pmp1	GGTG_03934
Development	Adhesion and hyphal regulator 1	GGTG_03357
	Hydrophobin	GGTG_03085, GGTG_02383, GGTG_06272, GGTG_07637, GGTG_04864, GGTG_08655
	DN24	GGTG_03133
	Cyclophilin	GGTG_01246, GGTG_06971
	Chitin synthase	GGTG_03012,GGTG_14037
	Phosphodiesterase	GGTG_01358, GGTG_01857, GGTG_03142, GGTG_06261, GGTG_10058, GGTG_11065
	Scytalone dehydratase	GGTG_01138
	Linoleate 9S-lipoxygenase	GGTG_02686
Plant cell wall degradation	Exopolygalacturonase	GGTG_09200, GGTG_05944
	Cellulase	GGTG_03106, GGTG_03626, GGTG_05117, GGTG_05722, GGTG_06476, GGTG_06689, GGTG_07662, GGTG_09001, GGTG_09705, GGTG_09705, GGTG_09799, GGTG_10590, GGTG_12435, GGTG_12679, GGTG_13103, GGTG_00778, GGTG_04874, GGTG_05674,

		GGTG_06629, GGTG_07637, GGTG_07808, GGTG_08211, GGTG_11851, GGTG_11952, GGTG_12375, GGTG_12397, GGTG_12670
	Xylanase	GGTG_02204, GGTG_02552, GGTG_03287, GGTG_04057, GGTG_05304, GGTG_05540, GGTG_08734, GGTG_09073, GGTG_11073, GGTG_13081, GGTG_13111, GGTG_13178,
	Xyloglucanase	GGTG_00152, GGTG_06573
	Aspartic protease	GGTG_03454, GGTG_12920
	Pectinesterase	GGTG_11671
Plant defense compounds	Gentisate 1, 2-dioxygenase-	GGTG_13883
	ABC transporter	GGTG_01796, GGTG_07693, GGTG_10893, GGTG_12859
	Laccase	GGTG_02239, GGTG_02417, GGTG_06952
	Glucosidase	GGTG_00113, GGTG_00803, GGTG_00708, GGTG_02652, GGTG_02124, GGTG_02179, GGTG_07024, GGTG_01694, GGTG_03156, GGTG_03193, GGTG_02476, GGTG_03211, GGTG_12383, GGTG_03453, GGTG_14198, GGTG_05010, GGTG_09671, GGTG_09709, GGTG_10943, GGTG_12367, GGTG_13224
